# Different *Candida parapsilosis* clinical isolates and lipase deficient strain trigger an altered cellular immune response

**DOI:** 10.3389/fmicb.2015.01102

**Published:** 2015-10-13

**Authors:** Renáta Tóth, Maria F. Alonso, Judith M. Bain, Csaba Vágvölgyi, Lars-Peter Erwig, Attila Gácser

**Affiliations:** ^1^Department of Microbiology, University of SzegedSzeged, Hungary; ^2^Aberdeen Fungal Group, Institute of Medical Sciences, University of AberdeenAberdeen, UK; ^3^Botany and Microbiology Department, King Saud UniversityRiyadh, Saudi Arabia

**Keywords:** *Candida parapsilosis*, phagocyte response, secreted lipase, co-infection, live cell imaging

## Abstract

Numerous human diseases can be associated with fungal infections either as potential causative agents or as a result of changed immune status due to a primary disease. Fungal infections caused by *Candida* species can vary from mild to severe dependent upon the site of infection, length of exposure, and past medical history. Patients with impaired immune status are at increased risk for chronic fungal infections. Recent epidemiologic studies have revealed the increasing incidence of candidiasis caused by non-albicans species such as *Candida parapsilosis*. Due to its increasing relevance we chose two distinct *C. parapsilosis* strains, to describe the cellular innate immune response toward this species. In the first section of our study we compared the interaction of CLIB 214 and GA1 cells with murine and human macrophages. Both strains are commonly used to investigate *C. parapsilosis* virulence properties. CLIB 214 is a rapidly pseudohyphae-forming strain and GA1 is an isolate that mainly exists in a yeast form. Our results showed, that the phagocyte response was similar in terms of overall uptake, however differences were observed in macrophage migration and engulfment of fungal cells. As *C. parapsilosis* releases extracellular lipases in order to promote host invasion we further investigated the role of these secreted components during the distinct stages of the phagocytic process. Using a secreted lipase deficient mutant strain and the parental strain GA1 individually and simultaneously, we confirmed that fungal secreted lipases influence the fungi's virulence by detecting altered innate cellular responses. In this study we report that two isolates of a single species can trigger markedly distinct host responses and that lipase secretion plays a role on the cellular level of host–pathogen interactions.

## Introduction

*Candida* species are the most common etiological agents of systemic fungal infections (Gácser et al., 2005). Although *Candida albicans* is the leading *Candida* species responsible for bloodstream infections, a significant increase has been reported in the number of fungal invasions caused by *non*-*albicans Candida* (NAC) species (Guinea, 2014). *C. parapsilosis* is one of the most frequent NAC species found in the hospital environment and currently is the number one cause of neonatal candidemia (Chow et al., 2012; Pammi et al., 2013; Guinea, 2014; Quindos, 2014). Besides its association with nosocomial infections in children, this species also threatens adult patients with diminished immunity (Nosek et al., 2009). Despite the emerging relevance of *C. parapsilosis*, relatively little is known about the immune responses induced by this species. GA1 and CLIB 214 are two distinct *C. parapsilosis* clinical isolates that are the most frequently used model strains for biological and molecular characterization studies (Gácser et al., 2005; Holland et al., 2014). *C. parapsilosis* GA1 is a bloodstream isolate obtained in Hamburg, Germany, and primarily appears in a yeast form and produces smooth colonies on agar (Trofa et al., 2008; Pryszcz et al., 2013). *C. parapsilosis* CLIB 214 (ATCC 22019) was isolated from the feces of a patient in Puerto Rico, and rapidly forms pseudohyphae producing a rough, concentric colony phenotype on agar (Laffey and Butler, 2005; Nosek et al., 2009). To our knowledge, no comparison has been made between the virulence of these commonly used laboratory type strains.

The phagocytic cells of the innate immune system play a central role in host defense against invading microbes, including fungi. The phagocytic process can be separated into four distinct stages: (1) phagocyte aggregation at the site of infection, (2) recognition of pathogen associated molecular patterns (PAMPs) via receptors, (3) internalization of the foreign particles, and (4) digestion of ingested agents through phagosome maturation and activation of hydrolytic enzymes (Lewis et al., 2013; Rudkin et al., 2013). We recently investigated the phagocytosis of *C. parapsilosis* CLIB 214 focusing on migration and engulfment of these cells by macrophages, and compared the results to that occurring with *C. albicans* and *C. glabrata* (Tóth et al., 2014b). In the present study, one of our primary aims was to compare and define the interactions of *C. parapsilosis* GA1 and CLIB 214 with host effector cells.

One well-described virulence factor that promotes the pathogenesis of invasive candidiasis is the secretion of hydrolytic enzymes, such as lipases and proteinases (Gácser et al., 2007; Horváth et al., 2012). Presumed roles of microbial secreted lipases during an infection include host cell adhesion, lipid digestion for nutrient acquisition and triggering of inflammatory cascades (Trofa et al., 2008; Nguyen et al., 2011; Tóth et al., 2014a). Secreted lipase encoding genes have been identified in *C. parapsilosis* (Gácser et al., 2007). In contrast with *C. albicans*, the *C. parapsilosis* genome includes only four putative secreted lipases (Butler et al., 2009; Nguyen et al., 2011). However, the deletion of only two of the four lipase encoding genes (*LIP1* and *LIP2*), resulted in significantly decreased virulence as determined in both *in vitro* and *in vivo* infection models (Nagy et al., 2011; Trofa et al., 2011). In addition, the deletion mutant strain lacking both *LIP1* and *LIP2* (*Cp*ΔΔ*lip*1 − ΔΔ*lip2*) formed less complex and thinner biofilms when compared to the *C. parapsilosis* GA1 parental strain (Gácser et al., 2007). Studies that used human peripheral blood mononuclear cell-derived macrophages (PBMC-DMs; Tóth et al., 2014a) and dendritic cells (DCs; Nagy et al., 2011) reported *Cp*ΔΔ*lip*1 − ΔΔ*lip2* strain to be less virulent, as both types of primary cells killed lipase mutant cells at a higher ratio than wild type cells. Furthermore, the lack of the secreted component, increased the pro-inflammatory cytokine and chemokine expression levels, thus led to stronger inflammatory response (Nagy et al., 2011; Tóth et al., 2014a). The decreased virulence of *Cp*ΔΔ*lip*1 − ΔΔ*lip2* was also reflected in the rate of reconstituted human tissue damage as the infected tissue was similar to the uninfected control and low LDH levels were measured (Gácser et al., 2007). Another study, using neonatal rats also confirmed these results, as low levels of organ fungal burdens were detected (Trofa et al., 2011).

Hence, the data show that secreted lipases play a role in *C. parapsilosis* virulence. Therefore, another major aim of the present work was to determine how secreted lipases influence the phagocytic process by examining the separate stages individually and if the presence of the wild type strain could complement the defective phenotype of *Cp*ΔΔ*lip*1 − ΔΔ*lip2* and thus influence the phagocyte response.

Thus, in this study we compared the phagocytosis of CLIB 214 by macrophages to that of GA1 and *Cp*ΔΔ*lip*1 − ΔΔ*lip2* cells using live cell imaging at defined stages of the phagocytic process: phagocyte migration, engulfment of fungal cells, and subsequent host cell damage. Additionally, co-infections with *Cp*ΔΔ*lip*1 − ΔΔ*lip2* and GA1 fungal cells were performed to determine whether secreted factors from wild type cells could complement the defects in the mutant yeasts.

## Materials and methods

### Preparation of *Candida parapsilosis* strains

Wild type *C. parapsilosis* CLIB 214 and GA1 clinical isolates and the *LIP1–LIP2* deficient strain (*Cp*ΔΔ*lip*1 − ΔΔ*lip2*) were maintained on YPD solid medium at 4°C and prepared for experiments as described in our previous work (Tóth et al., 2014b). Prior to experiments, the *Candida* strains were cultured overnight in liquid YPD medium (1% yeast extract, 2% glucose, 2% peptone) at 30°C with shaking at 200 rpm. The cells were collected and washed three times with PBS (phosphate buffered saline), counted and diluted to the final concentration of 1 × 10^8^/ml. UV-killed fungal strains were prepared using twenty exposures to 20 mJ/cm^−2^ UV. In order to differentiate between fungal cells after the co-infection, 1 mg/ml FITC (Sigma, Dorset, UK) and 50 μg/ml CFW (Sigma, Dorset, UK) were used to label yeasts. FITC was dissolved in dimethyl sulfoxide (DMSO) and added to 10^8^/ml cells suspended in 0.05 M carbonate-bicarbonate buffer (pH 9.6). CFW was dissolved in distilled water and added to 10^8^/ml yeasts in PBS. *C. parapsilosis* cells were stained at room temperature in the dark for 10 min, followed by washing steps three times with PBS and suspended in 1 ml 1X PBS. In dual yeast cell co-infection experiments, mixtures of GA1 and *LIP1–LIP2* yeast cells were used in which GA1 was labeled with FITC and the mutant with CFW. Experiments were simultaneously performed using GA1 yeast cells labeled with CFW and mutants with FITC. We found that there was no difference in our experiments whether the GA1 or lipase mutants were labeled with the alternate stain. Although we used both conditions in all experiments, we only reported out the condition where GA1 was labeled with FITC and the mutant with CFW.

### J774 mouse macrophage cell line preparation and staining

Dulbecco's modified Eagle's medium (DMEM; Lonza, Slough, UK) supplemented with 2 mM L-glutamine (Invitrogen, Paisley, UK), 10% fetal calf serum (FCS; Biosera, Ringmer, UK), and 200 U/ml penicillin/streptomycin (Invitrogen, Paisley, UK) was used in order to maintain the murine cell line. J774 cells were kept at 37°C, in the presence of 5% CO_2_ prior to and during the experiments. For live video microscopy, similarly to our previous report, 1.2 × 10^5^ macrophages were seeded on eight-well μ-slides (ibidi, Martinsried, Germany) and incubated at 37°C overnight prior to infection with *Candida* strains. For visualizing phagocytosis Lysotracker Red was used to label the acidic compartments of phagocytes. The original media was replaced with fresh, 300 μl pre-heated supplemented DMEM containing 1 μM Lysotracker Red DND-99 (Invitrogen, Paisley, UK) immediately before the experiments.

### Human PBMC-derived macrophage preparation and staining

For the isolation of human peripheral blood mononuclear cells (PBMCs), a standard protocol was used (Rudkin et al., 2013) with modifications, under approval from the institutional review board of the University of Aberdeen and the University of Szeged. Following isolation, PBMCs (7.5 × 10^5^ cell/ ml) were then plated on eight-well μ-slides (ibidi, Martinsried, Germany) and incubated at 37°C, 5% CO_2_ for 6–7 days in serum supplemented DMEM (Lonza, Slough, UK). Shortly before the experiment, similarly to the murine macrophage preparation, the media was replaced with 300 μl fresh, pre-heated supplemented and 1 μM Lysotracker Red DND-99 (Invitrogen, Paisley, UK) containing DMEM.

### Phagocytosis assay and live cell video microscopy

In order to examine the phagocytic processes post infection, a previously described and standardized protocol was used accordingly (Tóth et al., 2014b). Live and UV-treated *C. parapsilosis* CLIB 214, GA1, and ΔΔ*lip*1 − ΔΔ*lip2* cells were added to Lysotracker red DND-99-stained (Invitrogen, Paisley, UK; 1 μM) 4 × 10^5^ /ml J774 murine and 7.5 × 10^5^ /ml human PBMC-derived macrophages on eight-well μ-slides (ibidi, Martinsried, Germany) in 300 μl volumes immediately before the video capture was initiated. During individual infections, the effector/target ratio was 3:1, while in the case of simultaneous infections the ratio of 1.5 GA1: 1.5 lipase mutant: 1 macrophage was used. Images were captured over a 6 h period with a CCD camera attached to an UltraVIEW VoX Spinning disc confocal microscope (PerkinElmer, Massachusetts, USA) using a 40X oil immersion objective. For the comparison of the *C. parapsilosis* GA1 and CLIB 214 clinical isolates, over a 100 J774 macrophages were monitored individually and evaluated from at least four separate experiments—with 20–40 macrophages analyzed per experiment, dependent on frequency of macrophage–*Candida* interactions in the respective videos. From PBMC-DM experiments, 60 macrophages were tracked from three separate experiments with 15–20 macrophages analyzed per experiment due to cell size. For the *C. parapsilosis* GA1 and *Cp*ΔΔ*lip*1 − ΔΔ*lip2* comparison 250 macrophages were selected and monitored from five separate experiments, with 45–55 macrophages followed per experiment, dependent on frequency of macrophage–*Candida* interactions in the respective videos, in order to make clear assumptions from the co-infection studies.

For tracking and statistical analysis of both murine and human PBMC-derived macrophage migration Volocity 6.3 image analysis software (Improvision, PerkinElmer, Coventry, UK) was used. Similarly to our previous study, measurements included the macrophage migration toward *C. parapsilosis* cells, distribution of fungal cells per macrophages, prevalence of uptake events, engulfment time, average uptake of fungal cells by actively phagocytosing macrophages and post-ingestion rupture events of host cells.

High throughput analysis with Volocity software 6.3 allowed us to calculate the mean tract velocity of macrophages in the presence of different *C. parapsilosis* strains. The engulfment time was defined as the time difference between the establishment of the phagocyte-fungal cell contact and the phagocyte membrane enclosing around the fully ingested cell. Full engulfment events were determined when macrophages stretched toward fungal cells, the phagocyte membrane encircled around the yeast cell and the host cell regained its original shape. The prevalence of uptake represents the percentage of uptake events during defined time intervals. Individual events were defined by the starting point of the recognition of fungal cells by macrophages. The percentage of macrophages taking up specific numbers of fungal cell was referred to as distribution of fungal cells per macrophages. Average uptake is defined as the average number of fungal cells taken up by phagocytes that ingested at least one yeast cell over the whole incubation period. The percentage of dead macrophages relative to the visible macrophage population, that ingested at least one *C. parapsilosis* cell, represents post-ingestion macrophage rupture (PIR) events. Individual PIR events were defined as the disruption of membrane integrity that were visible up until the end of the 6 h of co-culturing period.

### LDH assay to determine host cell damage

To determine the concentration of LDH released by macrophages, culture supernatants were collected 6 h after *C. parapsilosis* infection. As a postive control, cells were treated with 1% Triton X-100 solution. For LDH detection, the Cytotoxycity Detection kit (Takara Bio Europe/Clontech, France) was used according to the user's manual. Basal LDH activity of human PBMC-derived macrophages may be due to the presence of undifferentiated monocytic cells or dead cells in the culture before the assay.

### Statistical analysis

Tracking of individual macrophages and high-throughput migration analysis was achieved by Volocity software 6.3. Extracted data were used to calculate the mean track velocity of phagocytes cultured with *Candida* strains.

For the comparison of the calculated mean values of each of the aspects, student's unpaired, two-tailed *t*-tests were used and confirmed by One-way ANOVA analysis followed by a Bonferroni's multiple comparison *post-hoc* tests. GraphPad Prism v 5.0 software was used to determine statistical significance. Significant differences were considered at *p*-values of ≤ 0.05. Data were pooled for migration analysis, engulfment time and LDH, thus SD-values are shown, percent phagocytosis, average uptake values and data for the fungal cell distribution/macrophages were evaluated as mean of means, thus SEM-values indicate error.

## Results

### Comparison of *C. parapsilosis* GA1 and CLIB 214 isolates

#### Macrophage migration differs for *C. parapsilosis* CLIB 214 and GA1 isolates

**Figure 1:**
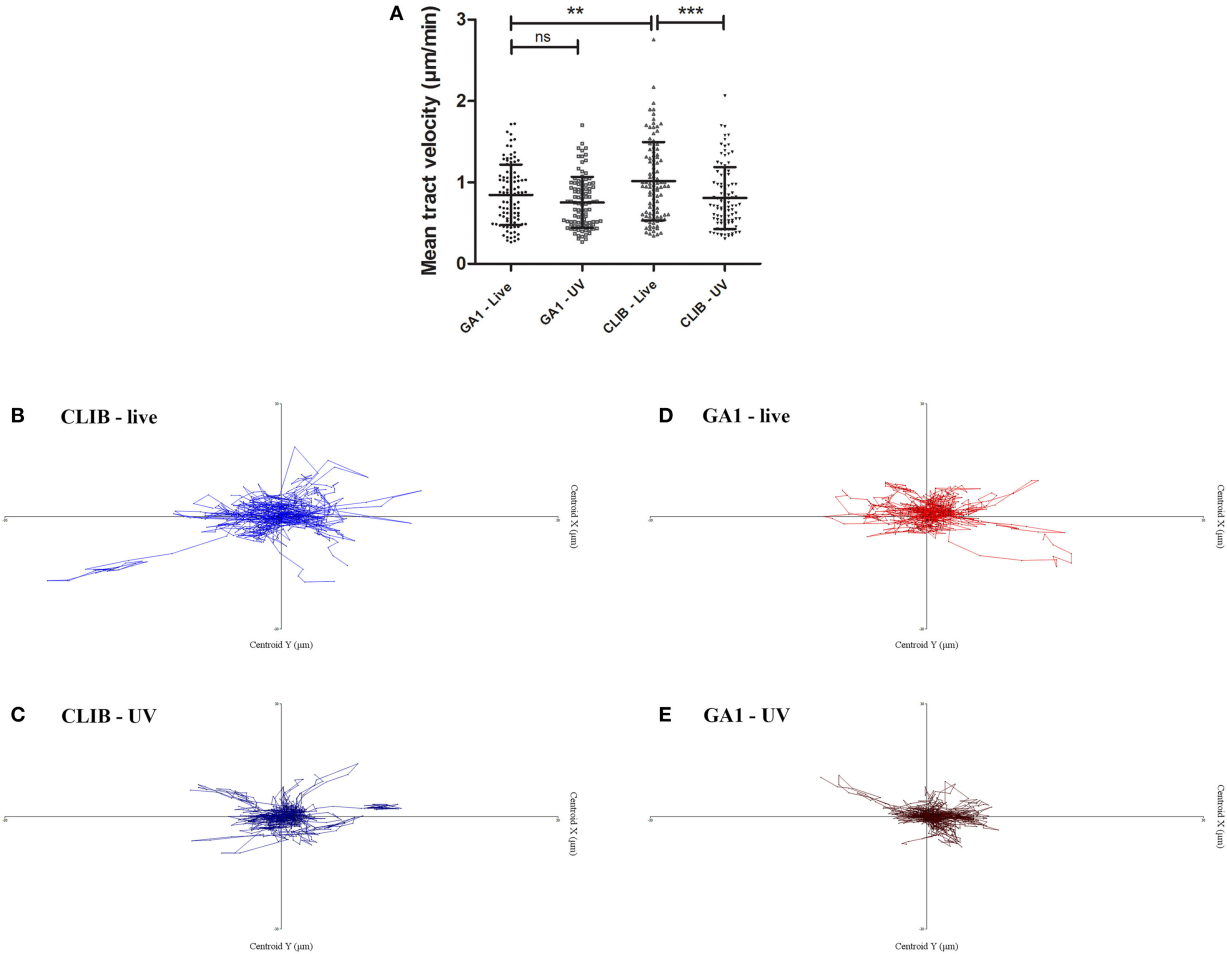
**Macrophage migration toward ***C. parapsilosis*** GA1 and CLIB 214**. J774 murine macrophages were challenged with live or UV-treated *C. parapsilosis* GA1 or CLIB 214 cells and were tracked individually using Volocity 6.3 software in defined time intervals. Mean track velocity values were calculated (mean ± SD) in μm/min **(A)**. Tracking diagrams show the movement and distances traveled by phagocytes relative to their starting position after culturing with live **(B)** or UV-killed **(C)** CLIB 214, and live **(D)** or UV-treated **(E)** GA1 cells. One-way ANOVA analysis with Bonferroni's multiple comparison test was used to determine statistical relevance. ^**^p < 0.01; ^***^p < 0.001.

Murine macrophages were challenged with live or UV-killed *C. parapsilosis* GA1 or CLIB 214 cells at an effector/target ratio of 1:3 and the phagocytic process was monitored by live cell video microscopy for 6 h (Lewis et al., 2012; Okai et al., 2015). We first examined the migration of macrophages toward the two distinct isolates. As the majority of uptake events were detected during the early stages post infection, macrophages were tracked during the first 45 min. Interestingly, the track velocity of J774 macrophages in response GA1 was significantly lower (mean ± SD; 0.85 ± 0.37 μm/min, Figure 1A) than to CLIB 214 (1.02 ± 0.48 μm/min). UV-killing of CLIB cells led to a significant reduction in the velocity of macrophage migration (0.81 ± 0.38 μm/min). In contrast, the difference between live and dead GA1 (0.75 ± 0.31 μm/min) was not significant, and the track velocity was similar to that of UV-irradiated CLIB yeast cells. These differences are also evident in the macrophage tracking diagrams (Figures 1B–E). The tracking diagrams show the movement and distances traveled by individual phagocytes relative to their starting position. The tracked murine macrophages appeared to travel shorter distances toward both UV-killed CLIB 214 and GA1 cells (Figures 1C,E) than toward live CLIB 214 and GA1 yeasts (Figures 1B,D).

#### CLIB 214 and GA1 exhibit altered engulfment dynamics during macrophage phagocytosis

**Figure 2:**
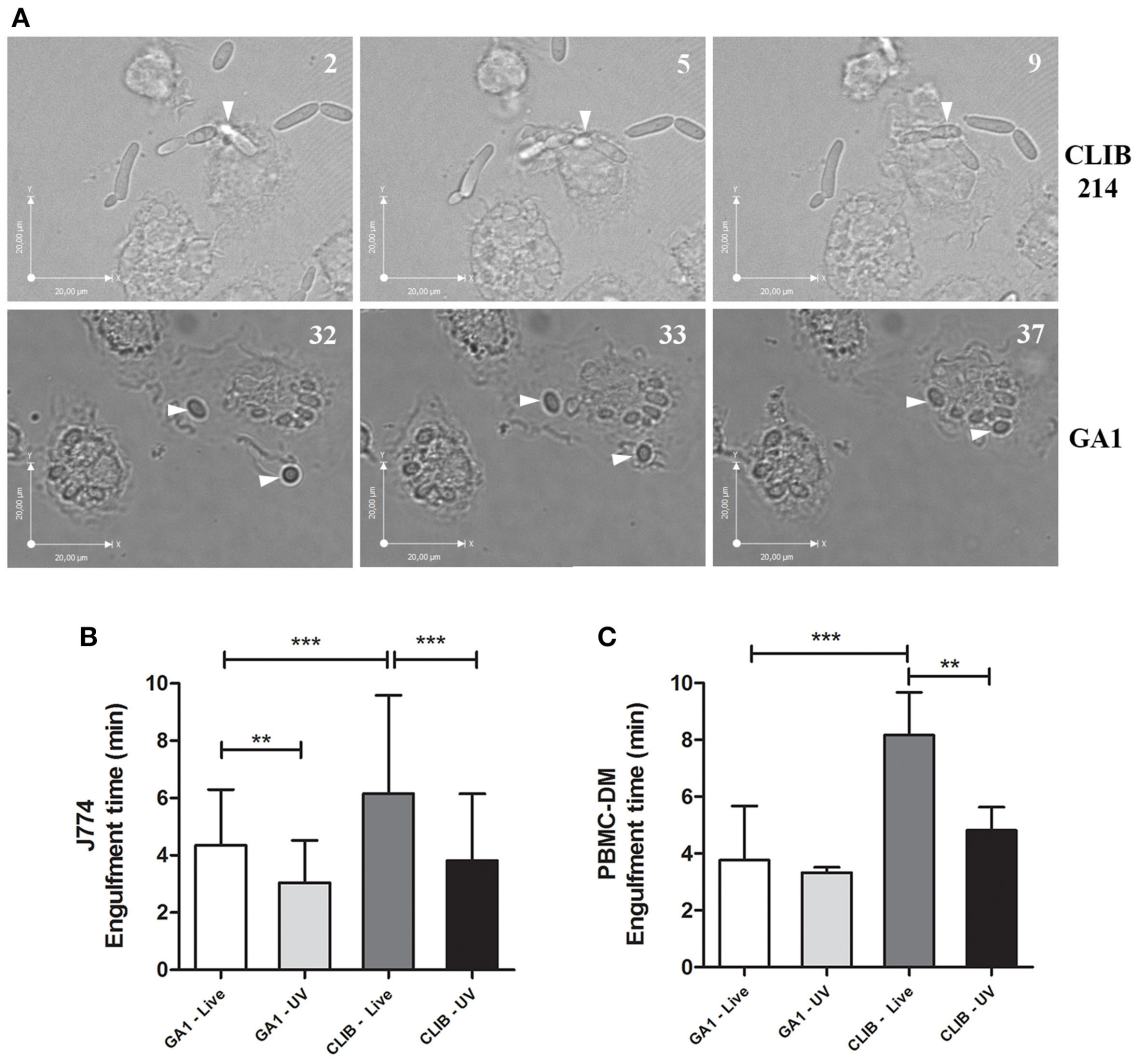
**Engulfment time of GA1 and CLIB 214 by macrophages**. Representative images show the full internalization of *C. parapsilosis* CLIB 214 **(A, upper panel)** and GA1 cells **(A, lower panel)** by human PBMC-derived macrophages. Numbers at the upper right corner of pictures show the time (minutes) of observed individual events. Arrows show the fungal cells internalized by phagocytes. Diagrams show the average time (mean-values + SD) in minutes required for the full ingestion of live or UV-treated GA1 and CLIB 214 cells by J774 **(B)** and PBMC-derived macrophages **(C)**. One-way ANOVA analysis with Bonferroni's multiple comparison test was used to determine statistical significance. Significant differences were considered at *p*-values ^**^p < 0.01; ^***^p < 0.001. Scale bar: 20 μm.

In this study we further aimed to differentiate between the time taken to internalize GA1 and CLIB 214 by murine and human PBMC-derived macrophages. Differences were observable in phenotype between the two isolates of *C. parapsilosis* as shown on Figure 2A, as rapid pseudohypha formation was detectable in CLIB 214 but not with the GA1 strain. At the early stage of infection, *C. parapsilosis* GA1 does not form pseudohyphae, only slightly elongated blastospores. Engulfment time is defined as the time from the first phagocyte-fungal cell contact and the macrophage fully enclosing the bound cell. According to our analyses, both J774 phagocytes and the human PBMC-derived macrophages required significantly less time to internalize GA1 yeast cells (mean ± SD; 4.36 ± 1.94 and 3.77 ± 1.9 min for J774 and PBMC-derived macrophages, respectively, Figures 2B,C) compared to CLIB 214 cells (6.16 ± 3.43 and 8.16 ± 6.41 min). Furthermore, UV-treatment significantly decreased the engulfment time by J774 macrophages of GA1 and CLIB 214 yeast cells (3.05 ± 1.47 and 3.82 ± 2.32 min, respectively; Figure 2B). A similar trend for the engulfment of UV-irradiated cells was shown when using human primary macrophages.

#### Uptake rates for the *C. parapsilosis* clinical isolates

**Figure 3:**
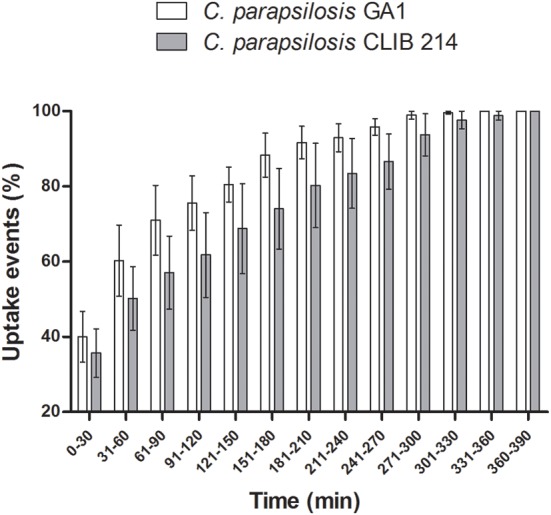
**Uptake kinetics of GA1 and CLIB 214**. Percentage of uptake events (mean ± SEM) following the co-incubation of J774 murine phagocytes with live *C. parapsilosis* GA1 or CLIB 214 cells. Individual events are associated with the beginning of fungal cell recognition by macrophages. Data were analyzed using Two-way ANOVA with Bonferroni's post-test. No differences were found after the evaluation.

The majority of fungal cells were phagocytosed during the first hour post-infection by murine macrophages (Figure 3). We observed no difference between the overall uptake of GA1 and CLIB 214 cells for both the murine and primary macrophages even though the primary cells showed a higher capacity for phagocytosis (Supplementary Figure 1). In general, the total uptake pattern of contribution of individual macrophages to overall uptake appeared to be similar for both clinical isolates and no statistically significant differences were observed in the average uptake of yeasts by both types of phagocytes (Supplementary Figure 2).

#### Host cell damage by CLIB 214 and GA1

**Figure 4:**
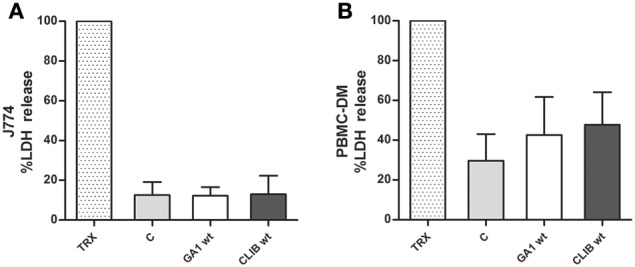
**Macrophage damage by GA1 and CLIB 214**. Host cell damage (mean + SD) was measured by the amount of lactate dehydrogenase (LDH) released by murine J774 **(A)** and human PBMC-derived **(B)** macrophages following incubation with live *C. parapsilosis* GA1 or CLIB 214 cells. In order to reach maximum LDH release Triton X-100 (TRX) was used. The supernatant of fungal-free cultured phagocytes was used as control “C”.

In order to examine the impact of yeast cells on the host effector cells, macrophage damage was measured by the amount of lactate dehydrogenase (LDH) released into supernatant after co-incubation with *C. parapsilosis* cells. Culture supernatant was collected 6 h after interaction with J774 phagocytes and human PBMC-derived macrophages, respectively. No differences were detected between the two *C. parapsilosis* clinical isolates in terms of their ability to damage either J774 (Figure 4A) or human macrophages (Figure 4B). LDH results were also confirmed by quantifying post-ingestion macrophage rupture events (Supplementary Figure 3).

### Role of fungal lipase secretion on phagocytosis

#### Comparison of *C. parapsilosis* GA1 and *Cp*ΔΔ*lip*1 − ΔΔ*lip*2 cells

**Figure 5:**
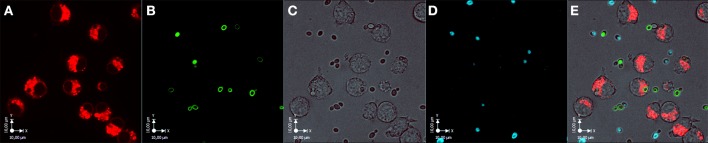
**Infection of murine J774 macrophages with ***C. parapsilosis*** GA1 and ***Cp***ΔΔ***lip***1 − ΔΔ***lip2*** cells, simultaneously**. The image series show four channels of the co-infection of J774 macrophages with GA1 and *Cp*ΔΔ*lip*1 − ΔΔ*lip2* cells. Channel **(A)**, Lysotracker red stained acidic compartments of phagocytic cells; channel **(B)**, fluorescein isothiocyanate (FITC) stained *Cp*ΔΔ*lip*1 − ΔΔ*lip2* yeasts; channel **(C)**, Bright-field image; channel **(D)**, Calcofluor white (CFW) stained GA1 cells; channel **(E)**, merged image. Images were taken immediately after infection (*T* = 0 min). Scale bar: 10 μm.

Murine macrophages were infected with *C. parapsilosis* GA1 or *Cp*ΔΔ*lip*1 − ΔΔ*lip2* individually in a ratio of 3:1. In the case of co-infection, GA1 and *Cp*ΔΔ*lip*1 − ΔΔ*lip2* cells were used in the ratio of 1:1, and the MOI was kept at 3:1 (representative image shown on Figure 5). In the mixed infections, yeast cells were either labeled with fluorescein isothiocyanate (FITC) or calcofluor white (CFW).

#### Migration toward wild type and *Cp*ΔΔ*lip*1 − ΔΔ*lip*2 mutant strain

**Figure 6:**
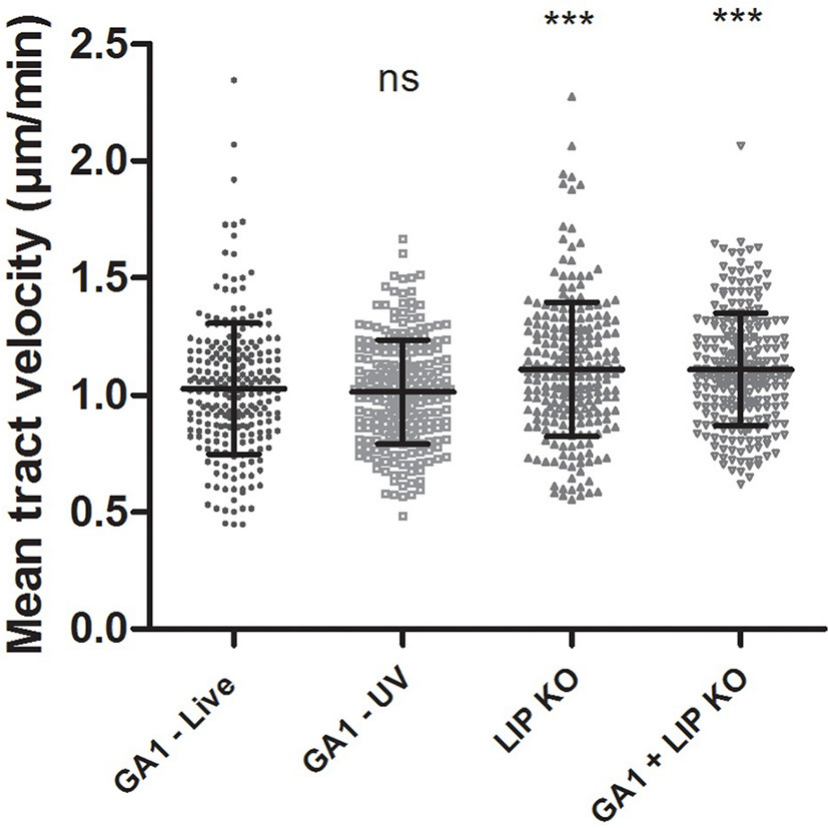
**Macrophage migration toward GA1, ***Cp***ΔΔ***lip***1 − ΔΔ***lip2*** or GA1, and ***Cp***ΔΔ***lip***1 − ΔΔ***lip2*** cells**. J774 murine macrophages were followed for 6 h after co-culturing with GA1 or *Cp*ΔΔ*lip*1 − ΔΔ*lip2* cells individually, and after GA1 and *Cp*ΔΔ*lip*1 − ΔΔ*lip2* simultaneous infections. Phagocyte mean track velocity values (mean ± SD) are shown in μm/min. One-way ANOVA analysis with Bonferroni's multiple comparison test was used to determine statistical relevance. Significance was determined relative to the wild type. Different gray symbols represent the mean tract velocity of individual macrophages. ^***^p < 0.001.

We assessed the migration of macrophages toward *Cp*ΔΔ*lip*1 − ΔΔ*lip2*, GA1 or mixed *Cp*ΔΔ*lip*1 − ΔΔ*lip2* and GA1 yeast cells during the first 45 min of co-incubation as above. The mean track velocity of J774 macrophages was significantly higher toward *Cp*ΔΔ*lip*1 − ΔΔ*lip2* (mean ± SD; 1.11 ± 0.28 μm/min; Figure 6) compared to GA1 (1.03 ± 0.28 μm/min). Interestingly, the macrophage migration was similar to that of *Cp*ΔΔ*lip*1 − ΔΔ*lip2* when both GA1 and *Cp*ΔΔ*lip*1 − ΔΔ*lip2* were present (1.11 ± 0.24 μm/min, Figure 6).

#### Differences in engulfment time of GA1 and *Cp*ΔΔ*lip*1 − ΔΔ*lip*2

**Figure 7:**
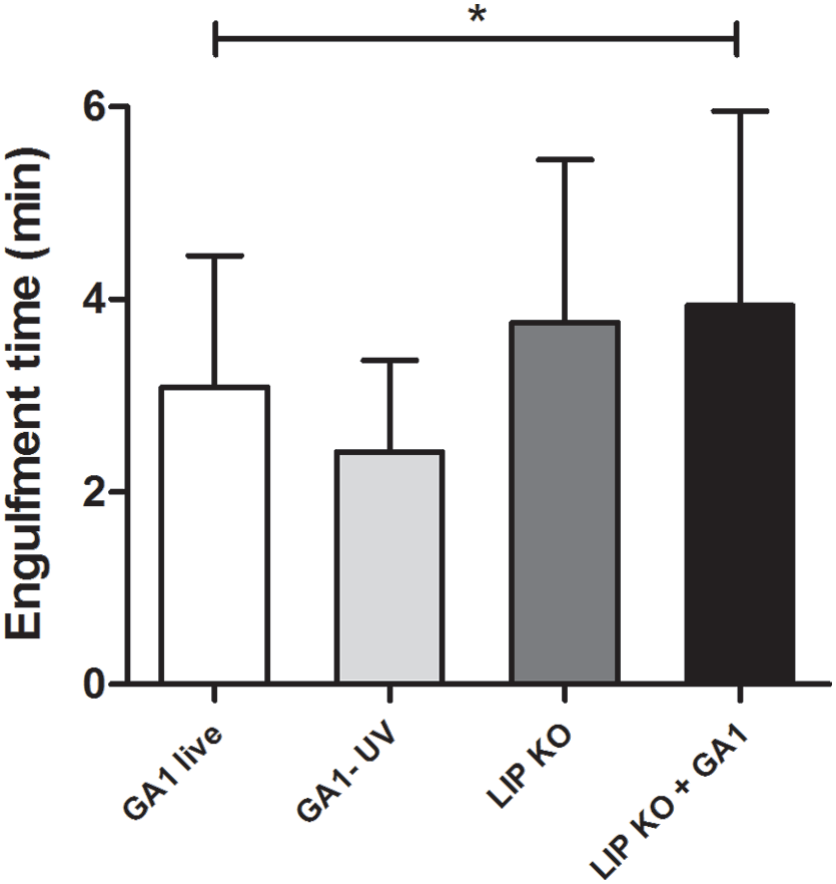
**Time required for the engulfment of GA1 and ***Cp***ΔΔ***lip***1 − ΔΔ***lip2*****. Diagrams show the average engulfment time (mean + SD in minutes) of live and UV-killed GA1 and LIP KO cells by J774 murine macrophages. Data were analyzed using One-way ANOVA with Bonferroni's post-test. Statistical significances were considered at *p*-values ^*^p < 0.05.

We also assessed whether there were differences in the engulfment times of *C. parapsilosis* GA1 and *Cp*ΔΔ*lip*1 − ΔΔ*lip2* cells by macrophages (Figure 7). Our results indicated, that more time was required for the full internalization of *Cp*ΔΔ*lip*1 − ΔΔ*lip*2 cells (mean ± SD; 3.76 ± 1.68 min; *p* = 0.07), in comparison with GA1 yeasts (3.09 ± 1.36 min). Mixed cultures of GA1 and lipase mutant yeasts had engagement times that were significantly increased relative to GA1 alone (3.948 ± 1.95 min, Figure 7), but were similar to the lipase mutants alone.

#### Differences in the phagocytosis of GA1 and *Cp*ΔΔ*lip*1 − ΔΔ*lip*2

**Figure 8:**
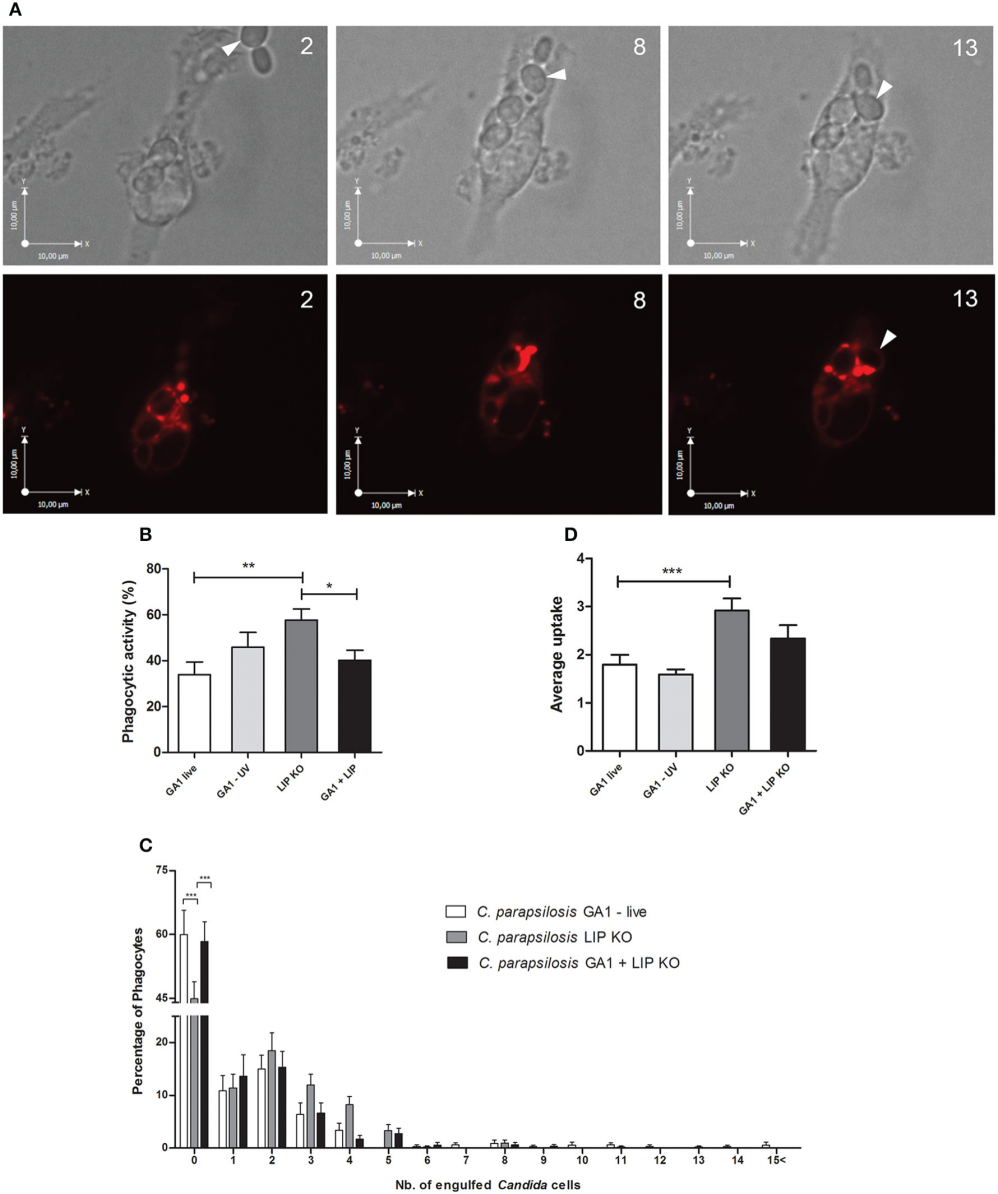
**Overall uptake of GA1 and ***Cp***ΔΔ***lip***1 − ΔΔ***lip2*** by J774 macrophages**. On Panel **(A)** bright field and fluorescent microscopic images show the internalization of *Cp*ΔΔ*lip*1 − ΔΔ*lip2* cells (arrows) by a J774 murine macrophage. Phagocyte was stained with Lysotracker Red. Numbers indicate the duration of time (min) from recognition until the complete ingestion of cells. Scale bar: 10 μm. Panels **(B–D)** represent the overall uptake of fungal cells showing phagocytic activity (mean + SEM; **B**), defined number of fungal cells taken up by phagocytes (mean + SEM; **C**) and average uptake (mean + SEM; **D**). Data for phagocytic activity and average uptake were analyzed using One-way ANOVA with Bonferroni's post-test. Two-way ANOVA analysis with Bonferroni's multiple comparison test (column to column comparison) was applied to determine significant differences between the distribution of fungal cells per macrophages. Significant statistical differences: ^*^p < 0.05; ^**^p < 0.01; ^***^p < 0.001.

We further examined the overall uptake of *C. parapsilosis* GA1 and the lipase deficient mutant strain by J774 macrophages. Representative images of *Cp*ΔΔ*lip*1 − ΔΔ*lip2* phagocytosis are shown on Figure 8A. In general, the overall uptake of mutant cells was more efficient than GA1 cells. Co-infection of phagocytes with both strains (Supplementary Video 1) led to less effective overall phagocytosis as shown here by the intermediate values. Our results showed that, significantly more J774 macrophages contributed to the uptake of lipase deficient cells (mean ± SEM; 57.62 ± 4.95%) compared to their interactions with GA1 yeasts (33.90 ± 5.53%, Figure 8B). There was a significant reduction in uptake of yeast cells in conditions in which both *Cp*ΔΔ*lip*1 − ΔΔ*lip2* and GA1 were present compared to the lipase mutant alone. A higher percentage of macrophages took up more than two or three *Cp*ΔΔ*lip*1 − ΔΔ*lip2* cells than GA1 cells (Figure 8C). The mean number of ingested yeast cells was significantly higher with *Cp*ΔΔ*lip*1 − ΔΔ*lip2* infection (mean ± SEM; 2.91 ± 0.25) compared to GA1 (1.79 ± 0.20) or co-infections with GA1 and lipase mutants (2.32 ± 0.20 Figure 8D). We found no difference in uptake kinetics for both types of strains either in case of single or co-infections (data not shown). No difference was detected between *Cp*ΔΔ*lip*1 − ΔΔ*lip2* or GA1 cell preference of the individual macrophages and no differences were observed in *Cp*ΔΔ*lip*1 − ΔΔ*lip2* or GA1 uptake, when both stains were present with the host cells (data not shown).

#### Macrophage damage by GA1 and *Cp*ΔΔ*lip*1 − ΔΔ*lip*2

**Figure 9:**
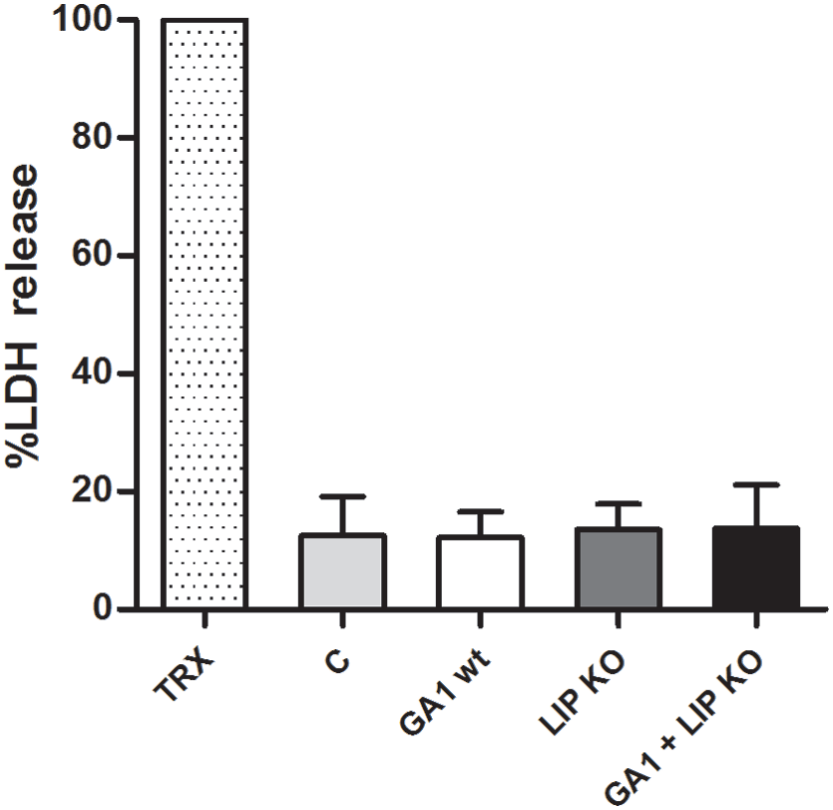
**Host cell damage by GA1 and ***Cp***ΔΔ***lip***1 − ΔΔ***lip2*****. LDH release (percentage of mean + SD) was measured to determine macrophage damage 6 h after co-incubation with *Cp*ΔΔ*lip*1 − ΔΔ*lip2*, GA1 or mixed *Cp*ΔΔ*lip*1 − ΔΔ*lip2* and GA1 yeast cells. TRX: Triton X-100 treated macrophages; C: fungal-free cultured phagocytes.

As a measure of host cell damage, LDH released by J774 phagocytes was measured from culture supernatant 6 h post-infection. Similar levels of LDH were detected with either individual; or mixed wild type and lipase deletion strains. Thus, the lack of *LIP1* and *LIP2* genes did not affect host cell damage (Figure 9). Similar observations were made when comparing the *C. parapsilosis* strains in terms of post-ingestion macrophage rupture events (data not shown).

## Discussion

In our previous study, we compared the phagocytic kinetics of three *Candida* species, *C. albicans, C. glabrata*, and *C. parapsilosis* CLIB 214 (Tóth et al., 2014b). Here we aimed to compare two well-described *C. parapsilosis* clinical isolates, GA1 and CLIB 214. In order to study the interaction between the innate immune system and *C. parapsilosis* clinical isolates on the cellular level, live cell imaging was used, which has previously been validated as a high-throughput image analysis tool for quantitative analysis (Bain et al., 2014; Okai et al., 2015). We compared phagocyte migration, engulfment rate, overall uptake of fungal cells, and subsequent host cell damage after challenging murine and human PBMC-derived macrophages with fungal cells. Our results showed similar overall uptake rates for GA1 and CLIB 214 by both types of macrophages as fungal strains were ingested during the early phases of the co-incubation; no differences were detected in phagocytic activity, uptake kinetics or average uptake. However, significant differences were found in terms of macrophage migration and engulfment time. Recent publications have already used macrophage migration to differentiate between pathogenic strains, as it refers to recognition capabilities of either pathogenic PAMPs or microbial signaling molecules (Lewis et al., 2012; Ifrim et al., 2014). Thus, host cell migration rates might indicate virulence properties of pathogenic microorganisms. Interestingly, we determined that the mean velocity of macrophages significantly decreased when they were incubated with GA1 yeast cells compared to CLIB 214. In addition, UV-treatment of either strain led to decreased phagocyte migration, although this difference was not significant for GA1. This change in migration might be caused by different amounts of fungal signaling molecules released into the medium, thus differences between the metabolic activities of the two isolates. However, further investigations are needed to confirm this theory. Furthermore, our data suggested that significantly more time was required for the uptake of CLIB 214 cells in contrast with GA1, and UV treatment significantly decreased engulfment time of both strains. As we previously reported, even though *C. parapsilosis* CLIB 214 cells are able to form pseudohyphae rapidly post-infection, no correlation was found between the engulfment time and the length of pseudohyphae (Tóth et al., 2014b). Thus, the difference in the engulfment time of GA1 and CLIB 214 might suggest that this is due to differences between the cell wall PAMPs of the two isolates, rather than differences in terms of shape and length. Results based on LDH release and post-ingestion macrophage rupture events revealed that both *C. parapsilosis* isolates examined contributed equally to host cell damage.

Even though, *C. parapsilosis* GA1 and CLIB 214 isolates were phagocytosed similarly, dissection of the phagocytic stages revealed various host cell responses. These altered responses might suggest differences in the cell wall structure or composition and fungal signaling molecules released by these strains. However, further investigations are needed for confirmation. As both of these clinical isolates are commonly used laboratory strains for molecular characterization, this information might be taken into consideration when studying gene function.

Secreted hydrolytic enzymes play a critical role in host invasion by fungi (Ghannoum, 2000; Borst and Fluit, 2003; Schaller et al., 2005). Secreted lipases of *Candida* species are associated with host cell adhesion, tissue damage and inflammatory response induction (Hube et al., 2000; Trofa et al., 2009). While *C. albicans* is known to have 10 lipase encoding ORFs, only two lipase genes (*LIP1*–*LIP2*) have been identified in *C. parapsilosis* (Hube et al., 2000; Stehr et al., 2004; Gácser et al., 2007). Both *C. parapsilosis* GA1 and CLIB 214 are known to produce extracellular lipases. Although, the previously established *C. parapsilosis* lipase deficient strain is only available on a GA1 background, thus when studying the function of lipases during an infection, we compared these strains only. The virulence properties of the *C. parapsilosis* lipase deficient strain (*Cp*ΔΔ*lip*1 − ΔΔ*lip2*) have already been investigated. According to our previous findings, the deletion mutant was killed more efficiently, however, phagocytosed similarly by macrophages when compared to the wild type (Tóth et al., 2014a). As shown previously, examining the component stages of the phagocytic process can provide insights into host–pathogen interactions on the cellular level and therefore, reveal differences in the recognition, overall uptake, and engulfment processes (Tóth et al., 2014b; Bain et al., 2015). In this study we examined the phagocyte response toward the *Cp*ΔΔ*lip*1 − ΔΔ*lip2* lipase mutant and the parental strain GA1. Numerous preceding studies have used co-infection methods to gain insight into virulence attributes (Silva et al., 2011; Bou Ghanem et al., 2012; Xu et al., 2014). Thus, in order to investigate whether the absence of extracellular lipase in the *Cp*ΔΔ*lip*1 − ΔΔ*lip2* yeast cells could be overcome with the secretion of lipases by wild type yeasts during an infection, murine macrophages were challenged with wild type and lipase mutant yeasts individually, and simultaneously. In general, the overall macrophage uptake of mutant cells was more effective, as a higher percentage of phagocytes ingested a greater number of *Cp*ΔΔ*lip*1 − ΔΔ*lip2* cells compared to the wild type yeasts. In correlation with the uptake rates, murine macrophage migration was increased toward mutant cells, indicating enhanced recognition of *Cp*ΔΔ*lip*1 − ΔΔ*lip2* compared to wild type cells. These results suggest that, the lack of secreted lipase results in decreased virulence on the cellular level. Although, our current results are in contrast with a previous study on *Cp*ΔΔ*lip*1 − ΔΔ*lip2* and GA1 phagocytosis comparison (Tóth et al., 2014a), this is not the first report revealing differences in *Cp*ΔΔ*lip*1 − ΔΔ*lip2* phagocytosis. Nagy et al. have used dendritic cells to study the virulence properties of *C. parapsilosis* secreted lipases and also found increased phagocytic efficiency (Nagy et al., 2011). Interestingly, the engulfment time of the mutant cells was prolonged. This data might suggest that, deficiency in lipase secretion might have led to modified cell wall structure or alterations in the expressed PAMPs or alterations in the level of other potential secreted signaling molecules. Similarly to our previous report on host cell damage by *Cp*ΔΔ*lip*1 − ΔΔ*lip2*, we found no difference in murine macrophage damaging capacity compared to the wild type.

In conditions when both fungal strains were present, a significant reduction was observed in overall phagocytosis of yeast cells compared to the lipase mutant alone. Thus, lipase deficiency can be partially restored with the presence of the wild type cells that further confirms the role of fungal extracellular lipases during host–pathogen interactions. No difference was detected in yeast cell preference when macrophages were incubated with both types of fungal cells further supporting our hypothesis on secreted component deficiency complementation. The presence of wild type secreted lipases might had a direct influence on fungal cell uptake regardless of the yeast cell types present in the media. However, co-infection with GA1 and lipase mutant yeast cells increased the engulfment time similarly to *Cp*ΔΔ*lip*1 − ΔΔ*lip2*. This result can be explained by the above mentioned hypothesis on an additional deficiency of *Cp*ΔΔ*lip*1 − ΔΔ*lip2* that cannot be rescued with the presence of secreted lipases. Interestingly, co-infection of macrophages with both strains led to significantly increased macrophage migration. As shown above, the presence of the *Cp*ΔΔ*lip*1 − ΔΔ*lip2* cells increased the average track velocity markedly. A possible explanation for this phenomenon is that in case of co-infection, the presence of the mutant cells might have unmasked a potential immune evasion ability in wild type cells. The presence of both strains simultaneously had no further effect on host cell damage compared to the wild type.

This study confirmed the role of secreted lipase in virulence, as lack of the secreted component resulted in decreased virulence by altering the dynamics of phagocytosis. Our results further showed that in terms of uptake, lipase deficiency is partially restored when the wild type strain is present, which further exemplifies the role of secreted lipases on phagocytosis.

In this report we have shown that two distinct isolates of a single species are able to trigger significantly different host responses and that fungal lipase secretion plays an important role in host–pathogen interactions on the cellular level.

### Conflict of interest statement

The authors declare that the research was conducted in the absence of any commercial or financial relationships that could be construed as a potential conflict of interest.

## Abbreviations in figures

LIP KO: indicates the *Cp*ΔΔ*lip*1 − ΔΔ*lip2* lipase deficient strain
TRX: represents Triton X-100.
