# Supplementary material for: Different Candida parapsilosis clinical isolates and lipase deficient strain trigger an altered cellular immune response
Source: Front Microbiol. 2015 Oct 13;6:1102. doi: 10.3389/fmicb.2015.01102 (PMC4602145; doi:10.3389/fmicb.2015.01102)
Supplement: Supplementary file 1 [file DataSheet1.DOC]

***Supplementary Material***

**Different *Candida parapsilosis* clinical isolates and lipase deficient strain trigger an altered cellular immune response**

**Renata Toth^1^, Maria Fernanda Alonso^2^, Judith Margaret Bain^2^, Csaba Vagvolgyi^1^, Lars-Peter Erwig^2^***^†^***, and Attila Gacser^1^***^†^**

^1^Department of Microbiology; University of Szeged; Szeged, Hungary

^2^Aberdeen Fungal Group, Institute of Medical Sciences, University of Aberdeen, Aberdeen, United Kingdom

*^†^:* These authors have contributed equally to this work.

*** Correspondence:** Attila Gacser: gacsera@gmail.com

## Supplementary Figures


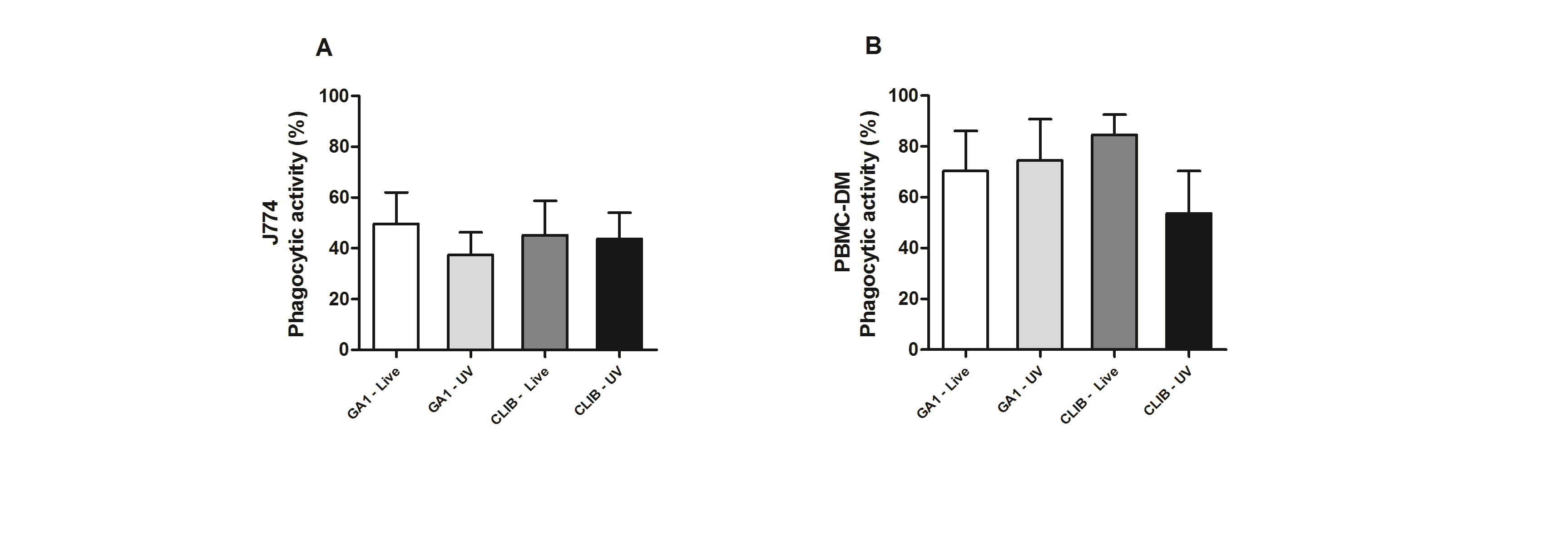


**Supplementary figure 1. Percent phagocytosis of *C. parapsilosis* GA1 and CLIB 214 cells.** The percent of actively phagocytosing macrophages (mean + SEM) are shown after incubation with GA1 and CLIB 214 live and UV- treated cells relative to the observed phagocyte population. Equal amount of murine macrophages (A) and human PBMC-derived macrophages (B) contributed to the uptake of all strains used for the experiment.


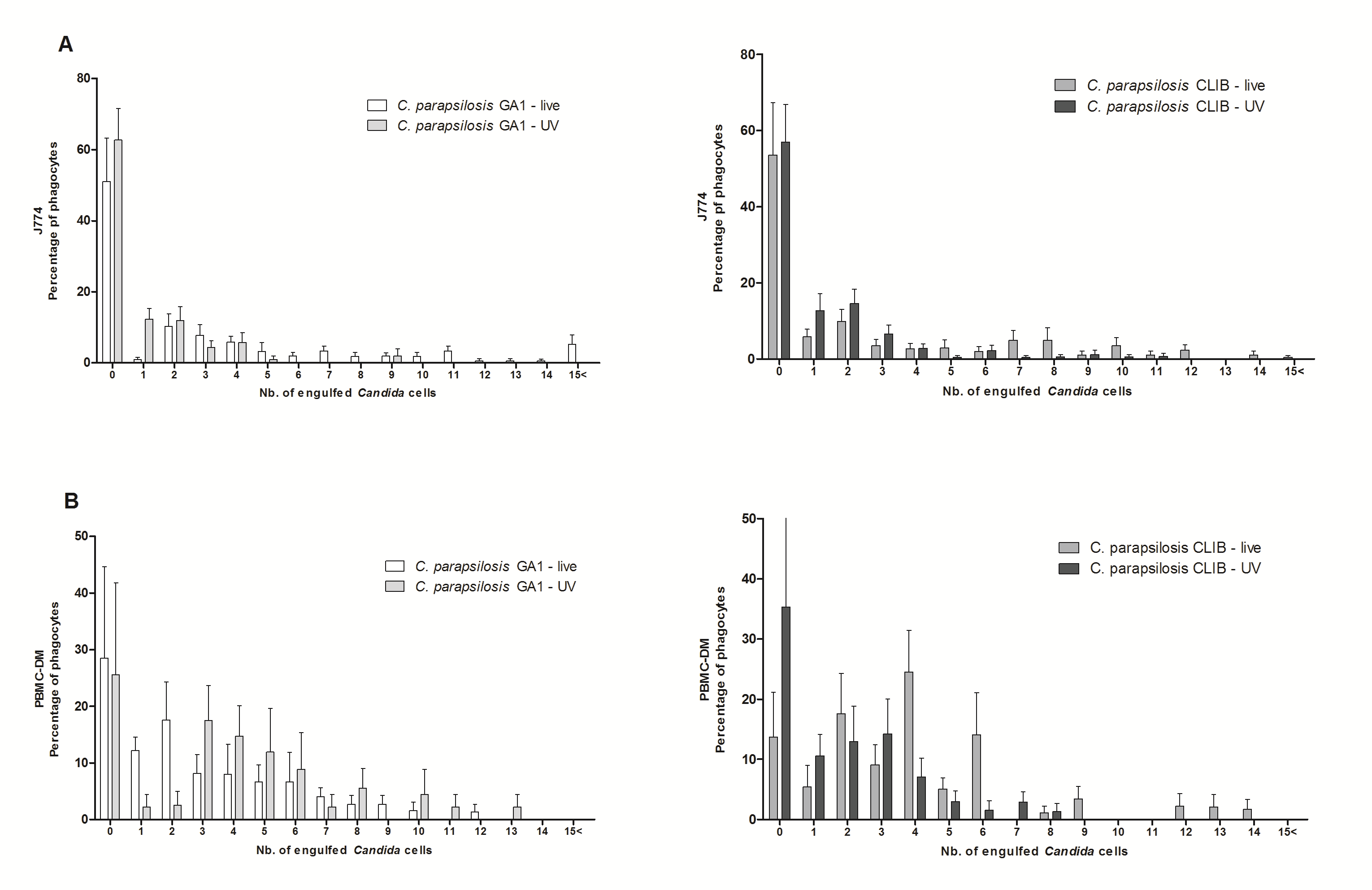


**Supplementary figure 2. Uptake rates of individual macrophages.** Diagrams show the percentage (mean + SEM) of J774 phagocytes (A) and human PBMC-derived macrophages (B) taking up defined numbers of fungal cells. Phagocytes were challenged with *C. parapsilosis* GA1 and CLIB 214, live and UV killed cells at a MOI of 3:1. Interactions were monitored for 6 hours.


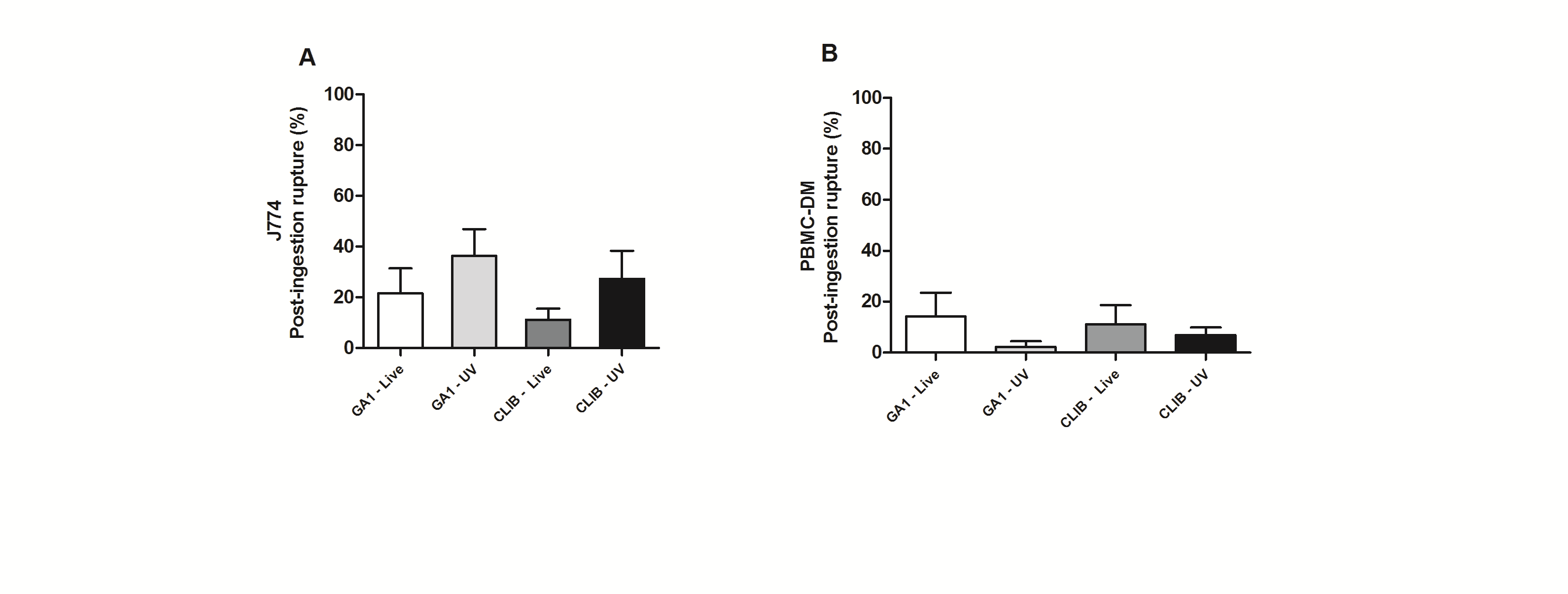


**Supplementary figure 3. Post-ingestion macrophage rupture events.** Percentage of macophage rupture events (mean + SEM) was determined and compared after incubation of murine phagocytes (A) and human PBMC-derived macrophages (B) with *C. parapsilosis* GA1 and CLIB 214 live and UV killed cells. All examined fungal strains showed similar host cell damaging capacity.

1. **Supplementary videos**

**Supplementary video 1. Co-infection of murine macrophages with GA1 and *CpΔΔlip1- ΔΔlip2.*** Representative video of the first hour of J774 macrophage infection with GA1 and *CpΔΔlip1- ΔΔlip2* simultaneously. GA1 cells were stained with FITC, *CpΔΔlip1- ΔΔlip2* with calcofluor white, and macrophages were labeled with Lysotracker red. Scale bar: 10µm.
