# Supplementary figures and images for: Different Candida parapsilosis clinical isolates and lipase deficient strain trigger an altered cellular immune response
Source: Front Microbiol. 2015 Oct 13;6:1102. doi: 10.3389/fmicb.2015.01102 (PMC4602145; doi:10.3389/fmicb.2015.01102)

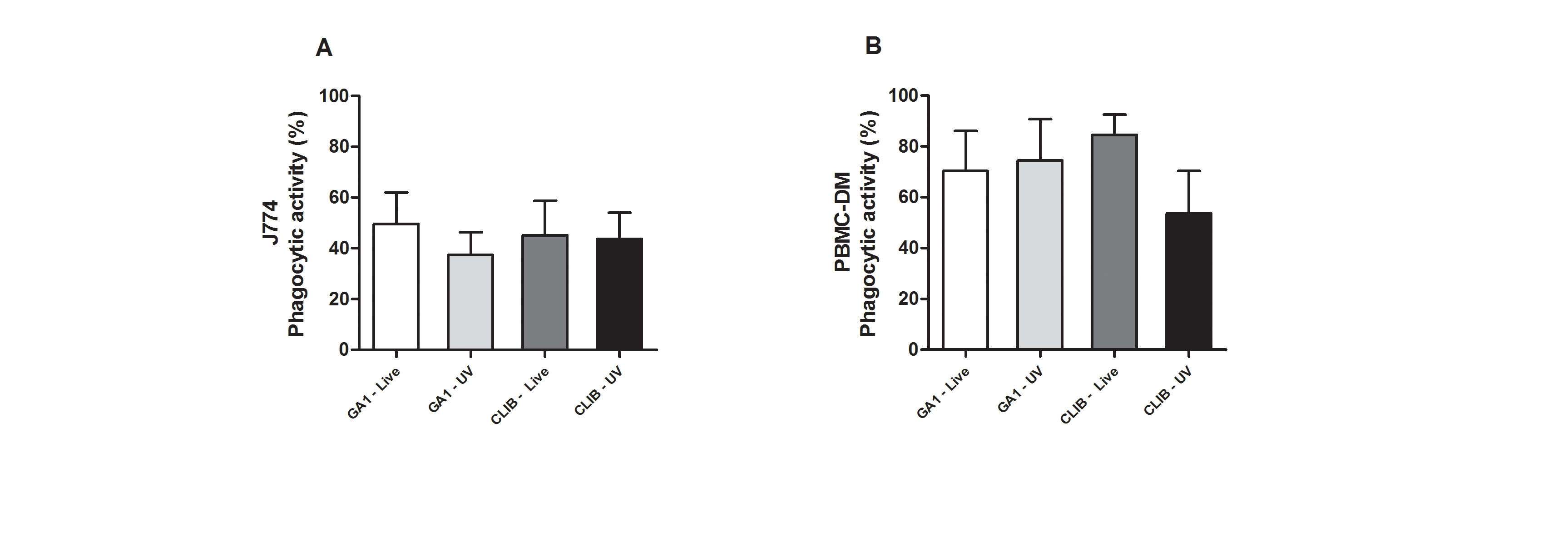

Supplement: Supplementary file 2 [file Image1.TIF]

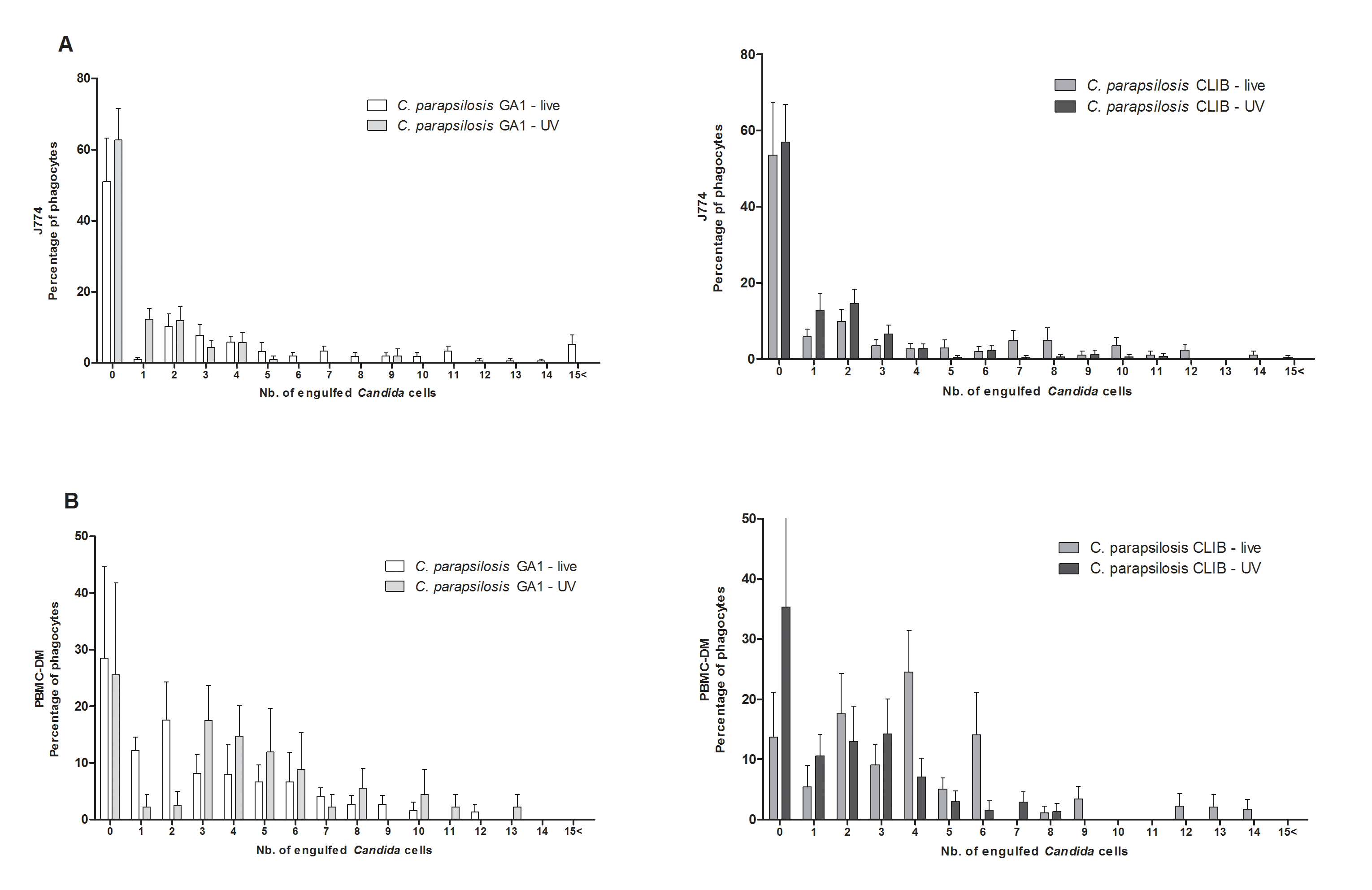

Supplement: Supplementary file 3 [file Image2.TIF]

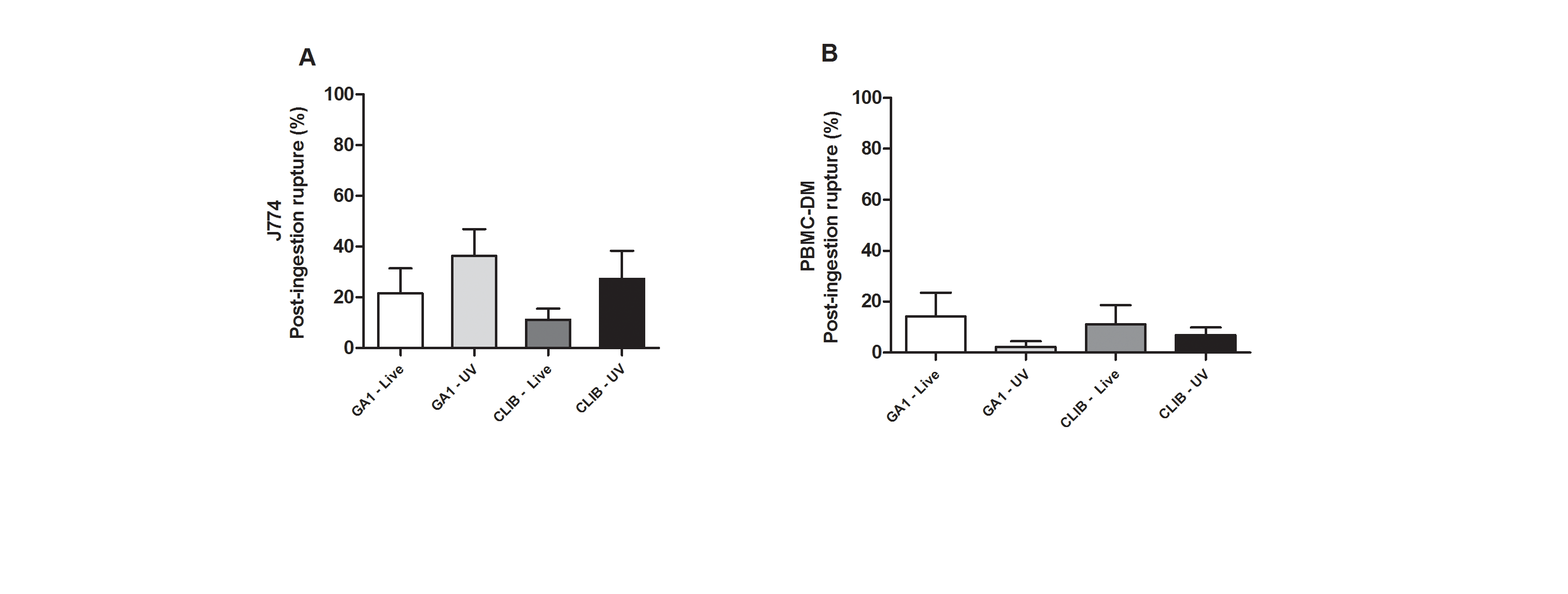

Supplement: Supplementary file 4 [file Image3.TIF]
